# Birdsongs alleviate anxiety and paranoia in healthy participants

**DOI:** 10.1038/s41598-022-20841-0

**Published:** 2022-10-13

**Authors:** E. Stobbe, J. Sundermann, L. Ascone, S. Kühn

**Affiliations:** 1 Lise Meitner Group for Environmental Neuroscience, Max Planck Institute for Human Development, Lentzeallee 94, 14195 Berlin, Germany; 2 Neuronal Plasticity Working Group, Department of Psychiatry and Psychotherapy, University Medical Center Hamburg-Eppendorf, Martinistr. 52, 20246 Hamburg, Germany

**Keywords:** Human behaviour, Environmental impact

## Abstract

The present study investigated the effect of urban (traffic noise) vs. natural (birdsongs) soundscapes on mood, state paranoia, and cognitive performance, hypothesizing that birdsongs lead to significant improvements in these outcomes. An additional goal was to explore the differential impact of lower vs. higher diversity of the soundscapes by manipulating the number of different typical traffic sounds or songs of different bird species within the respective soundscapes. In a randomized online experiment, N = 295 participants were exposed to one out of four conditions for 6 min: traffic noise low, traffic noise high, birdsong low, and birdsong high diversity soundscapes. Before and after the exposure, participants performed a digit-span and dual n-back task, and filled out depression, anxiety, and paranoia questionnaires. The traffic noise soundscapes were associated with a significant increase in depression (small effect size in low, medium effect size in high diversity condition). Concerning the birdsong conditions, depression exclusively decreased after exposure to the high diversity soundscape (small effect size). Anxiety and paranoia significantly decreased in both birdsong conditions (medium effect sizes). For cognition, no effects were observed. In sum, the present study suggests that listening to birdsongs regardless of diversity improves anxiety, while traffic noise, also regardless of diversity, is related to higher depressiveness. Moreover, for the first time, beneficial, medium-sized effects of birdsong soundscapes were demonstrated, reducing paranoia. Overall, the results bear interesting implications for further research, such as actively manipulating soundscapes in different environments or settings (e.g., psychiatric wards) and testing their effect on subclinical or even clinical manifestations of anxiety and paranoia.

## Introduction

The impact of environmental influences on psychological well-being and cognition in humans have for a long time been neglected in traditional psychology. At present, human living environments are changing drastically. According to the UN 2007 was a turning point for humankind as for the first time the majority of the global population lived in urban areas^1^. Until 2050, it is estimated that 68% of the world population will be living in cities^2^. In Europe the urbanization rate is already as high as 75%. Urbanization coincides with increasing rates of mental illness. An earlier review from 2005 came to the conclusion that about 30% of the incidence in schizophrenia may be attributed to urban factors in interaction with genetic liability and social adversity^3^. A meta-analysis^4^ shows a link between the increase of schizophrenia incidence and the increase in urbanicity, highlighting the fact that the risk for schizophrenia in the most urban environment was estimated to be 2.37 times higher than in the most rural environment. In a study investigating environmental factors known to trigger paranoia^5^, it was shown that urban cyclers commonly report to experience at least one state paranoia reaction, reported on a paranoia scale, in response to what the authors call an interpersonal threat situation. This was caused by the presence of potentially dangerous traffic participants, such as motor vehicle drivers. According to Ellet et al.^5^ the key environmental factors known to trigger paranoia include threat and ambiguity, which may be more often found in urban compared to natural environments. A recent review on depression and urbanicity reported mixed results, however with the majority of studies suggesting an elevated risk of depression in more (vs. less) urbanized areas^6^. Concerning mood and anxiety disorders, a review on studies conducted in Europe concludes that most studies showed elevated risks for mood/anxiety symptoms when comparing some (albeit not all) of the urban to rural areas^7^. In sum, there is hence accumulating evidence that living in urban areas is related to worse mental health outcomes.

Contrary to the negative effect of urban environments on mental health, a recent study shows that increasing access to total and usable green space within the neighborhood can decrease anxiety/mood disorder treatment counts. This demonstrates the service that urban green spaces may provide for general mental health and well-being^8^. Another study, which looked at the perceived sensory dimensions (PSD) relevant for attentional and stress recovery in green spaces, found that natural environments which are serene, provide refuge, and are rich in species diversity, as well as are perceived as highly ‘natural’, are rated as most restful^9^. These effects are often explained by two predominant theories. Stress reduction theory (SRT) posits that landscapes, containing vegetation, water and other aspects that provide benefits for survival, help to moderate and reduce states of arousal and negative thoughts and thus reduce the psychological and physiological symptoms of stress^10^. Similarly, Attention Restoration Theory (ART) states that stimuli from natural sources restore cognitive function by reducing attention demands of the endogenous attention system^11^.

Experimental research in the field has so far predominantly studied visuo-spatial aspects of the environment concerning mental health effects, such as by using photographs, videos, slideshows, or other visual stimuli. Other sensory modalities have been much less studied^12^. Man-made (urban) soundscapes (the so-called anthrophone) can constitute constant stressors that may impair cognitive function and well-being. Urban noise contains salient stimuli that likely trigger an alert physiological and psychological state. Corroborating this notion, a systematic review on traffic noise exposure found consistent evidence for an association between traffic noise and depression, as well as cognitive decline^13^. On the other hand, natural soundscapes, which are typically characterized by birdsongs, wind, or water^12^, could be an important source of attention-restorative and stress-ameliorating effects, as they might be implicitly associated with a safe and vital natural environment. As documented by a narrative review, birdsongs, water-, and wind-sounds have been shown to be perceived as pleasant, and to have beneficial effects on mood, arousal levels, and cognitive performance^12^. Importantly, in several studies that have reported positive effects of birds on human well-being, higher species diversity was a relevant factor^14^, perhaps because it may indicate the vitality or intactness of natural spaces. In a study across 26 countries conducted by Methorst et al.^15^, the authors established a relationship between species diversity of birds within a region and self-reported life satisfaction of residents of those regions. Remarkably, it was found that a 10% increase in bird species diversity raises life-satisfaction approx. 1.53 times more than a proportional rise in income^15^.

In a randomized controlled experimental study using auditory stimuli, hypotheses derived from ART and SRT were tested. Van Hedger et al.^16^ compared a nature vs. city soundscape condition, whereby the former enhanced participants’ performance in a dual n-back and digit span task, but did not improve mood. The present study broadly builds on the study by van Hedger et al.^16^ but adds the factor of diversity to the soundscapes. In addition to mood and cognition (dual n-back and digit span task), the present study additionally focusses on state paranoia, as this is a very prominent symptom in psychosis which can be measured in a change-sensitive manner^17^. Furthermore, state paranoia has been shown to increase in response to traffic noise (e.g., building-site noise)^18^. However, investigating in how far natural vs. urban auditory stimuli might influence this symptom category has, to our knowledge, not systematically been studied yet. The present study thus addressed the following hypotheses: (1) birdsong (vs. traffic noise) soundscapes have a beneficial effect on mood and paranoia; (2) birdsong (vs. traffic noise) soundscapes have a beneficial effect on cognitive performance. Furthermore, it was investigated whether greater (vs. lower) diversity of bird species or noise sources within the soundscapes would be a relevant factor, modulating the effects. The outcomes (mood, paranoia, cognitive performance) were each measured before and after soundscape exposure. For each soundscape type a low vs. high diversity version was created. This resulted in a between 2 (*type*: birdsongs vs. traffic noise) × 2 (*diversity*: low vs. high) × within 2 (*timepoint:* pre vs. post) randomized experimental design.

## Results

### Perception of soundscapes

The results of the MANOVA, revealed significant effects of *type* (*F*(3, 276) = 78.6, *p* < 0.001, η_p_^2^ = 0.461), *diversity* (*F*(3, 276) = 3.16, *p* = 0.025, η_p_^2^ = 0.033), as well as *type* × *diversity* (*F*(3, 276) = 2.66, *p* = 0.028), suggesting that all of these factors as well as their interaction had a significant impact on the perception of soundscapes (i.e., ratings on monotony/diversity, beauty, and pleasantness).

Univariate follow-up ANOVAs revealed for the factor *type*, that it only significantly affected beauty (*F*(1, 278) = 168.8, *p* < 0.001, η_p_^2^ = 0.378) and pleasantness perceptions (*F*(1, 278) = 182.3, *p* < 0.001, η_p_^2^ = 0.396), but not monotony/diversity *type* (*F*(1, 278) = 0.06, *p* = 0.812, η_p_^2^ = 0.000). Concerning the factor *diversity*, it only significantly affected the monotony/diversity ratings *type* (*F*(1, 278) = 6.21, *p* = 0.013, η_p_^2^ = 0.022), but not the other rating dimensions of beauty (*F*(1, 278) = 0.84, *p* = 0.361, η_p_^2^ = 0.003) or pleasantness (*F*(1, 278) = 0.58, *p* = 0.448, η_p_^2^ = 0.002). Finally, concerning the interaction *type* × *diversity*, there were no significant effects on monotony/diversity (*F*(1, 278) = 0.75, *p* = 0.387, η_p_^2^ = 0.003) nor beauty (*F*(1, 278) = 2.84, *p* = 0.093, η_p_^2^ = 0.010), but on pleasantness (*F*(1, 278) = 5.36, *p* = 0.021, η_p_^2^ = 0.019).

For statistical details on the post-hoc tests, see Table 1, (for descriptive data on the qualitative sound ratings see Supplementary Table 1). Low vs. high diversity conditions differed significantly from one another on the according monotony/diversity rating dimension, albeit with a small effect size. This effect was attributable to a significant small rating difference between the low and high traffic noise conditions; however, the low vs. high bird conditions were not perceived as significantly different concerning monotony/diversity. This speaks for only a partially successful manipulation of diversity. Both beauty and pleasantness were always perceived as significantly higher for the birdsong conditions in any given comparison with the traffic noise conditions (all *p* < 0.001), with large effect sizes.

**Table 1:** Comparison of soundscapes concerning beauty, pleasantness, and diversity/monotony ratings in the total sample.

DV	Conditions	*t*(df)	*p*-value	Cohen’s *d*
**Monotony/diversity**				
*Type* traffic noise vs. birdsongs		0.50 (278)	0.619	0.06
*Diversity* low vs. high		− 2.59 (280)	0.010*	− 0.21
Traffic noise low vs. traffic noise high		− 2.38 (146)	0.019*	− 0.39
Traffic noise low vs. birdsong low		− 0.81 (142)	0.418	− 0.13
Traffic noise low vs. birdsong high		− 1.89 (153)	0.060	− 0.31
Traffic noise high vs. birdsong low		1.69 (125)	0.094	0.30
Traffic noise high vs. birdsong high		0.45 (136)	0.657	0.07
Birdsong high vs. birdsong low		1.18 (131)	0.240	0.21
**Beauty**				
*Type* traffic noise vs. birdsongs		13.1 (276)	0.000***	1.54
*Diversity* low vs. high		− 0.21 (280)	0.831	− 0.03
Traffic noise low vs. traffic noise high		1.77 (147)	0.079	0.28
Traffic noise low vs. birdsong low		− 7.65 (142)	0.000***	− 1.24
Traffic noise low vs. birdsong high		− 8.75 (146)	0.000***	− 1.38
Traffic noise high vs. birdsong low		− 10.8 (125)	0.000***	− 1.79
Traffic noise high vs. birdsong high		− 11.6 (136)	0.000***	− 1.98
Birdsong high vs. birdsong low		0.61 (131)	0.546	0.11
**Pleasantness**				
*Type* traffic noise vs. birdsongs		13.4 (280)	0.000***	1.59
*Diversity* low vs. high		− 0.34 (280)	0.732	− 0.04
Traffic noise low vs. traffic noise high		2.14 (147)	0.036*	0.35
Traffic noise low vs. birdsong low		− 7.60 (142)	0.000***	− 1.28
Traffic noise low vs. birdsong high		− 9.50 (152)	0.000***	− 1.51
Traffic noise high vs. birdsong low		− 9.78 (125)	0.000***	− 1.74
Traffic noise high vs. birdsong high		− 11.7 (136)	0.000***	− 2.00
Birdsong high vs. birdsong low		1.15 (131)	0.252	0.20

*p < .05; ***p < .001.

#### Mood and paranoia

Univariate analyses of variance revealed no baseline differences across the groups in the outcome variables at baseline for depression (*F*(3, 291) = 0.31, *p* = 0.820), or anxiety (*F*(3, 291) = 0.31, *p* = 0.821). However, across the groups there were significant differences in state paranoia (*F*(3, 291) = 3.34, *p* = 0.020). For details (exact group differences and descriptive baseline data), see Supplementary Table 2.

**Figure 1:**
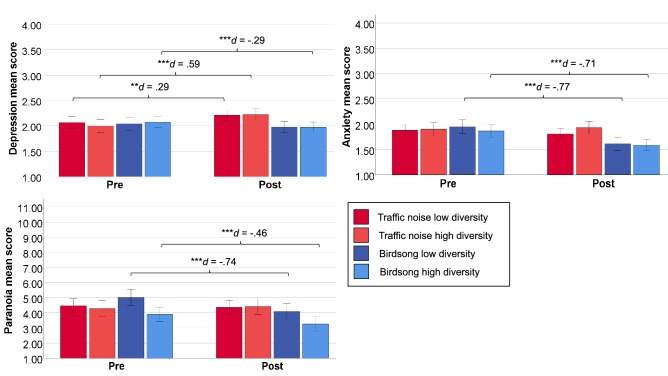
Within-group changes in mood and paranoia for all variables of interest. Y-axes have been formatted to reflect the possible data range. The interpretation of scores corresponds to the Likert-scale of the respective measure. Between-group differences (at baseline) and exact descriptives (means and standard deviations) for pre- and post-tests can be found in Supplementary Table 2. Paired t-test statistics for changes within groups in mood (anxiety, depression) and paranoia can be found in Supplementary Table 3.

For depression, there was a significant *time* effect (*F*(1, 291) = 4.51, *p* = 0.035, *η*^2^_*partial*_ = 0.015), suggesting overall changes in depressive states from pre-to-post. The soundscape *type* × *time* interaction was significant (*F*(1, 291) = 32.1, *p* < 0.001, *η*^2^_*partial*_ = 0.099), the triple interaction *type* × *diversity* × *time* was non-significant (*F*(1, 291) = 1.52, *p* = 0.217, *η*^2^_*partial*_ = 0.005), suggesting that *diversity* was not a modulating factor of the differential pre-post-change by *type*. Additional analyses were run to check the robustness of the effects i.e., controlling for state paranoia age, and positive symptoms which differed significantly (paranoia) or at trend (p < 0.10; age, positive symptoms) at baseline as covariates, and excluding cases who did not type in at least one digit of the auditory codeword at the end of the soundscape correctly (= listening compliance check). The results of these analyses were comparable to the above reported ones. Post-hoc examination of the effects by computing within group dependent t-tests revealed that depressive symptoms significantly increased within both the low diversity urban soundscape (*T*(1, 82) = 2.64, *p* = 0.010, *d* = 0.29) and high diversity urban condition (*T*(1, 68) = 4.88, *p* < 0.001, *d* = 0.59), whereas there were differential effects in the birdsong conditions with no change in the low diversity condition (*T*(1, 62) =  − 1.49, *p* = 0.142, *d* =  − 0.19) but a significant decrease in the high diversity condition (*T*(1, 60) =  − 2.57, *p* = 0.012, *d* =  − 0.29) (see Fig. 1).

For anxiety, there was a significant *time* effect (*F*(1, 291) = 39.9, *p* < 0.001, *η*^2^_*partial*_ = 0.121), suggesting overall changes in state anxiety from pre-to-post. The soundscape *type* × *time* interaction was significant (*F*(1, 291) = 30.1, *p* < 0.001, *η*^2^_*partial*_ = 0.094), the triple interaction *type* × *diversity* × *time* was non-significant (*F*(1, 291) = 0.15, *p* = 0.704, *η*^2^_*partial*_ = 0.000), suggesting that *diversity* was not a modulating factor of the differential pre-post-change by *type*. Additional analyses were run to check the robustness of the effects (for details see previous section), which revealed that the findings were not altered by entering the covariates and/or excluding cases with incorrect codeword (failed listening compliance check). Post-hoc within-group t-tests for anxiety revealed that there were no effects within both traffic noise conditions (low diversity: *T*(1, 82) =  − 1.37, *p* = 0.174, *d* =  − 0.15; high diversity: *T*(1, 68) = 0.49, *p* = 0.629, *d* = 0.06), whereas there were significant declines in both birdsong conditions (low diversity: *T*(1, 62) =  − 6.13, *p* < 0.001, *d* =  − 0.77; high diversity: *T*(1, 60) =  − 6.32, *p* < 0.001, *d* =  − 0.70) (see Fig. 1).

As paranoia levels differed significantly at baseline between the groups, instead of a 2 × 2 × 2 repeated measures ANOVA approach, univariate ANCOVA, with paranoia at baseline as covariate and post-test paranoia as outcome, predicted by *type* and *diversity* as factors, was computed. *Type* was a significant factor explaining post-test paranoia (*F*(1, 290) = 45.5, *p* < 0.001, *η*^2^_*partial*_ = 0.070), *diversity* was non-significant (*F*(1, 290) = 0.50, *p* = 0.480, *η*^2^_*partial*_ = 0.002), as was the interaction *type* × *diversity* (*F*(1, 290) = 0.18, *p* = 0.670, *η*^2^_*partial*_ = 0.001). Finally, for paranoia there were no changes in the traffic noise conditions (low diversity: *T*(1, 82) =  − 0.55, *p* = 0.583, *d* =  − 0.06; high diversity: *T*(1, 68) = 0.67, *p* = 0.507, d = 0.08), but significant decreases in both birdsong conditions (low diversity: *T*(1, 62) =  − 5.90, *p* < 0.001, *d* =  − 0.74; high diversity: *T*(1, 60) =  − 4.11, *p* < 0.001, *d* =  − 0. 46) (Fig. 1).

#### Cognition

Univariate analyses of variance revealed no baseline differences across the groups in the cognition outcome variable at baseline for the forward digit span two-error maximum length (TE_ML) measure (*F*(3, 291) = 0.45, *p* = 0.720), or the according backward digit span measure *(F*(3, 291) = 0.15, *p* = 0.962). Univariate analyses of variance also revealed no baseline differences across the groups in the cognition outcome variable at baseline for the dual n-back measure *d* prime (*F*(3, 291) = 1.01, *p* = 0.388).

Concerning the two-error maximum length digit span forward, there was no significant *time* effect (*F*(1, 281) = 1.1, *p* = 0.298, *η*^2^_*partial*_ = 0.004). The *time* × soundscape *type* interaction effect was also not significant (*F*(1, 281) = 0.2 *p* = 0.662, *η*^2^_*partial*_ = 0.001), as well as the *time* × *type* × *diversity* triple interaction (*F*(1, 281) = 1.1, *p* = 0.296, *η*^2^_*partial*_ = 0.004). The same null-finding emerged for the according backward digit span measure: *time* (*F*(1, 281) = 0.30, *p* = 0.582, *η*^2^_*partial*_ = 0.001), *time* × soundscape *type* (*F*(1, 281) = 1.04, *p* = 0.308, *η*^2^_*partial*_ = 0.004) and *time* × *type* × *diversity* (*F*(1, 281) = 1.2, *p* = 0.278, *η*^2^_*partial*_ = 0.004). For the n-back task total performance parameter, there were also consistent null-findings. There was no significant effect of *time* (*F*(1, 287) = 0.37, *p* = 0.543, *η*^2^_*partial*_ = 0.001) or *time* × soundscape *type* (*F*(1, 287) = 0.23, *p* = 0.635, *η*^2^_*partial*_ = 0.001) nor *time* × *type* × *diversity* (*F*(1, 287) = 1.12, *p* = 0.279, *η*^2^_*partial*_ = 0.004). Additional analyses were run to check the robustness of the effects i.e., controlling for state paranoia age, and positive symptoms which differed significantly (paranoia) or at trend (p < 0.10; age, positive symptoms) at baseline as covariates, and excluding cases who did not type in at least one digit of the auditory codeword at the end of the soundscape correctly (= listening compliance check). The results of these analyses were comparable to the above reported ones. At last, an aggregation of both task scores into a composite *z*-score, which is an established practice in the field^16^, did not yield a significant result either.

## Discussion

The present study built up a previous study by van Hedger et al.^16^, who demonstrated that a natural vs. urban soundscape was related to better cognitive outcomes, but failed to demonstrate significant effects on mood. In the present study, birdsongs were contrasted with traffic noise, whereby a novel diversity factor (low vs. high number of birdsongs or traffic noise sources in the soundscapes that participants were exposed to) was introduced, and effects on paranoia were additionally tested (for the rationale see “Introduction” section).

Opposed to van Hedger et al.^16^ in the present study only effects on mood (depression, anxiety) and paranoia, but not on cognition (dual n-back, digit span task), were found. Traffic noise soundscapes generally aggravated depressive states (small effect in low diversity, moderate effect in high diversity condition), whereby these soundscapes were also perceived as significantly different in terms of diversity in subjective ratings, which were conducted as a manipulation check at the end of the study. Exclusively the highly diverse birdsong soundscape decreased depressive states (small effect size). Generally, the birdsong conditions were not rated as significantly different in terms of diversity. Concerning anxiety, traffic noise soundscapes had no effect, whereas both birdsong soundscapes significantly alleviated anxiety (medium effect sizes). Finally, the traffic noise soundscapes had no effect on paranoia, whereas again both birdsong soundscapes significantly lowered it (medium effect sizes).

The beneficial effects of birdsongs in particular concerning mood and attention restoration have been previously observed^20^. Mood recovery (e.g., after a stressor) or beneficial mood effects have repeatedly been reported for exposure with natural sounds^21,22^. The present study thus confirms prior findings. Moreover, to the best of our knowledge, beneficial effects of natural soundscapes on state paranoia are shown for the very first time. This finding might be explained in several ways. Birdsongs might be implicitly associated with a vital natural environment, divert attention away from (internal and external) stressors, or could signal the absence of acute threat. Urban soundscapes on the other hand might trigger socio-evaluative concerns, involuntarily direct attention resulting in perceived loss of control and hence alter vigilance to potential threats which are processes proposed to elicit paranoia. However, somewhat contradicting the latter notion, the traffic noise soundscapes as used in the present study did not increase paranoia. Possibly, by adding human voices to the audio file, this effect could have been evoked. Human voices may more readily activate interpersonal sensitivity, to which a central role has been ascribed in the emergence and maintenance of paranoia^23^. Generally, classical learning paradigms (conditioning) might provide a framework to explain restorative nature effects. Hereby, first an unconditioned positive response occurs in reaction to nature, which gets later retrieved by similar natural cues, and can later generalize to an abstract level whereby even more abstract cues (e.g., words) may trigger the original response^24^.

Neither urban (traffic noise) nor natural (birdsong) soundscapes had any effect on cognitive performance, which, at first glance, seems to contradict a previous study, which implemented the same cognitive tests (i.e., digit span and dual n-back)^16^. An explanation for the null effect could be the degree to which the administration of the tasks was controlled. The current study was performed online, practice was restricted to two blocks (i.e., in a laboratory setting, participants can often train for as long as they wish/need), and hence there was little control over the degree to which subjects understood the task correctly (albeit visual inspection of the raw data did not reveal severe deviations from an expected performance). In addition, the online as opposed to a laboratory situation does not allow for controlling context variables or systematically manipulating baseline levels of stress or fatigue^21^. Besides the highlighted methodological issues with respect to the cognitive outcomes of the current study, it could be debated whether such an effect of exposure to nature on cognitive performance as measured by executive functioning tasks really exists on a population level. Support for the existence of this effect originally comes from a study in which mere viewing of pictures from nature improved cognitive performance^11^. This was subsequently replicated with auditory exposure to nature in the form of soundscapes using the same cognitive tasks that were also administered in the current study^16^. Summing up this debate there hence seems to be preliminary support for the existence of such an effect on a population level, however future meta-analyses about this effect should be carried out in order to clarify the existence of the effect.

Concerning the fact that there was no difference between the high and low diversity soundscapes of birdsongs with respect to the effects on mood and paranoia it should be mentioned that in a study by Methorst et al.^15^ where bird species diversity in a given region was shown to be related to reported life-satisfaction in that region, the authors suggest two explanations which are also relevant in the light of the afore-mentioned result. It is concluded that the multisensory experience of birds can be a crucial factor for diversity of birdsongs to have an effect on life-satisfaction. Yet another explanation is that beneficial landscape properties in fact drive the effects, promoting both bird diversity and people’s life-satisfaction independently of one another. In the current study it might be the case that merely listening to a more diverse birdsong soundscape did not communicate the same multisensory experience than it does when people are experiencing bird diversity in a real-life situation. Furthermore, the appreciation of diversity might rely on certain knowledge or expertise, resulting in a benefit only for experienced listeners. Potentially, our sample did include mostly lay people concerning bird listening, which could partially explain the result with respect to the diversity variable (i.e., the soundscapes were not rated as significantly different from one another in terms of monotony/diversity). Future studies investigating this topic should aim to include some kind of expertise measurement in order to control for this factor.

## Limitations

One limitation of the present study is the numerically higher percentage of males relative to females (albeit non-significant), which is a typical problem encountered in online studies. Future studies should stratify the subgroups by sex, such as to balance the sample in this regard. Furthermore, the manipulation of low vs. high diversity of the soundscapes was only partially successful. Namely, although the soundscape composition followed a logical rationale in this regard, and the subjective diversity rating the urban soundscapes significantly differed in the traffic noise soundscapes, this was not the case for the birdsong conditions. To assure stronger contrasts, and hence to be able to test the diversity hypothesis more aptly concerning enhanced beneficial effects, the contrast between the soundscapes needs to be further enhanced in future studies. In addition, mixed conditions such as urban soundscapes containing birdsongs would be a highly interesting research target, as this could more readily reflect daily life exposure situations.

It remains to say that the current study made use of a non-clinical sample opening the debate if the observed effects can also be generalized to people diagnosed with high levels of e.g., paranoia. Taking into account the continuum hypothesis, which states, that psychotic symptoms which are seen in patients can also be observed in non-clinical populations, it might be the case that both populations share a mechanism by which symptoms can be relieved or improved. This remains speculative, nevertheless the current study can be seen as a pilot, exploring the existence and potential magnitude of effects, feasibility, and safety for a future transfer to a more vulnerable clinical sample. Future research could adopt the current design and investigate the effects of birdsong exposure on paranoia within a clinical sample. Such research can potentially result in low threshold environmental interventions to reduce distress in e.g., psychiatric wards or other clinical settings. In the sense of conditioning (see above), associations with natural environments might divert attention away from psychological stressors or signal the absence of acute threat. Future experiments could aim to explore if paranoia does in fact not decrease after exposure to threatening natural environments, such as the wilderness, or situations which signal the acute presence of threat, such as natural disasters.

Moreover, it is important to highlight the fact, that the results of the current study cannot provide any clarification concerning the sustainability or replicability (e.g., by repeated exposure) of the effect birdsongs can have on mood and paranoia. Future research should aim to test such effects in a longitudinal and/or repeated exposure study design. Yet another limitation in the current study is the lack of a neutral control sound condition which would enable the interpretation of results with respect to a neutral condition instead of the mere comparison between a traffic condition and a birdsong condition. The use of such a neutral control group within a similar design as implemented by the current study would be a great addition for future research. Finally, although the instructions required participants to set their audio system loudness to 80%, still subjectively perceived loudness of the soundscapes could constitute a confounding factor.

### Conclusion

The present study provides evidence for the beneficial effects of birdsongs on mood (depression, anxiety) and paranoid symptoms, with the latter being shown for the first time. On the other hand, the negative effects of traffic noise were only confirmed concerning depressive symptoms. The manipulated low vs. high diversity of the soundscapes did not have a significant effect, which might in part be explained by no perceived subjective differences concerning monotony vs. diversity. Further replication in vulnerable, elevated risk- or clinical groups could be of interest to assess the magnitude of effects given pre-existing symptoms and cognitive performance deficits. In case of replication, using birdsongs as ‘soothing’ background soundscape could open interesting new possibilities in psychiatric hospitals or other therapeutic settings.

## Methods

### Power calculation and study registration

A power calculation was conducted for an interaction effect (repeated measures ANOVA), in G*Power 3.1.9.7 with f = 0.10, α = 0.05, power = 0.90, 4 groups, correlation between repeated measures r = 0.60, resulting in a minimum required total sample size of N = 288 (n = 72 per group). According to the general rule of thumb for Cohen’s f statistic, f ≥ 0.10 < 0.25 is a small effect, f ≥ 0.25 < 0.40 is a medium effect, and, f ≥ 0.40 a large effect (see Cohen, 1988)^25^. We opted for a small effect size as a similar study as ours, conducted by van Hedger et al.^16^, also using a repeated-measures ANOVA data analysis approach, reported interaction effects *type* ([2] natural vs. urban soundscapes) by *time* ([2] pre-to-post exposure) on mood, whereby Cohen’s d for negative affect was between 0.36 and 0.40. These results were non-significant, as the study was underpowered for detecting small effects. The interaction effects observed concerning cognition in that paper, applying the same tests as in the present paper, were large (d between 0.71 to 0.76). Since we were interested in detecting effects on mood and to study yet unknown effects on state paranoia, we opted for and intermediate effect size between small and medium.

The study was pre-registered at aspredicted.org (study name: “Sounds_Online”, trial identifier: #67702, https://aspredicted.org/d5j7j.pdf) on 06/04/2021.

### Recruitment and in- and exclusion criteria

The study was programmed using Inquisit 5^26^ (https://www.millisecond.com) and accordingly run on the Millisecond server. Participants were recruited from the crowdsourcing platform Prolific and received 10€ reimbursement for their full participation. Adult individuals were pre-screened on Prolific (i.e., visibility of the study only for candidates with a suited profile) concerning fluent German language skills (as this was the study language), having no diagnosed lifetime mental illness, and having no hearing difficulties. Pre-screened individuals could then access the study, where in- and exclusion criteria were checked further. This included no regular substance or drug intake, no suicidal thoughts, or tendencies, and availability of headphones for the purpose of the study.

### Study procedure

After providing informed consent, sociodemographic information was assessed, including education, income, and further variables, which were assessed for potential additional or exploratory analyses, but for the sake of conciseness are not reported in this paper. Psychosis liability was assessed. For an according overview on sample characteristics, see Table 2. Hereafter, pre-test assessments were conducted, including an assessment of mood (depression, anxiety), paranoia, the digit-span, and n-back tasks. Participants were randomized to one of four sound conditions: (1) low diversity traffic noise soundscape n = 83, (2) high diversity traffic noise soundscape n = 69, (3) low diversity birdsong soundscape n = 63, or (4) high diversity birdsong soundscape n = 80, (for details on the stimuli, see “Stimuli” section). The soundscapes each lasted for exactly 6 min. Participants were instructed to set their audio system volume to 80% (which was piloted with members of our research unit beforehand and deemed to be an optimal average volume) and to listen to the sounds until the end, when participants were required to continue by clicking with their mouse. Participants were told that a code, consisting of two spoken digits (in German), would be audible towards the end of the sound presentation, which they were required to type in correctly afterwards. This was implemented to assure listening-compliance and attention. After the sound presentation, the pre-test measures were repeated. Finally, several items to assess perceived sound quality, including beauty, pleasantness, and monotony (vs. diversity) were presented.

**Table 2:** Descriptive sample data and between-group differences for socio-demographic variables.

Variable	Traffic noise low n = 83	Traffic noise high n = 69	Birdsong low n = 63	Birdsong high n = 80	Inferential statistics
Age mean (SD)	27.0 (7.48)	25.5 (7.10)	26.5 (6.30)	28.7 (7.72)	*F*(3, 293) = 2.53, *p* = 0.057
Sex: % male (n)	55% (45)	64% (44)	71% (45)	54% (43)	*X*^2^(3, 293) = 6.04, *p* = 0.110
**School degree**^**1%**^** (n)**					
None	1.20% (1)	2.90% (2)	6.30% (4)	3.80% (3)	*X*^2^(9, 293) = 6.04, *p* = 0.285
Low	11.0% (9)	11.6% (8)	15.9% (10)	7.60% (6)	
Middle	13.4% (11)	21.7% (15)	17.5% (11)	10.1% (8)	
High	73.4% (61)	63.8% (44)	60.3% (38)	78.5% (62)	
**Net income % (n)**					
< 1.250€	48.8% (40)	43.5% (30)	41.3% (26)	40.5% (32)	*X*^2^(21, 293) = 21.3, *p* = 0.443
1.250–1.749€	11.0% (9)	7.20% (5)	15.9% (10)	16.5% (13)	
1.750–2.249€	7.30% (6)	8.70% (6)	15.9% (10)	8.90% (7)	
2.250–2.999€	12.2% (10)	8.70% (6)	12.7% (8)	10.1% (8)	
3.000–3.999€	2.40% (2)	10.1% (7)	4.80% (3)	3.80% (4)	
4.000–4.999€	3.70% (3)	0.00% (0)	1.60% (1)	1.30% (1)	
> 5.000 €	2.40% (2)	2.90% (2)	3.20% (2)	2.40% (3)	
Not wish to answer	12.2% (10)	18.8% (13)	4.80% (3)	15.2% (12)	
CAPE positive symptoms freq. score^2^ mean (SD)	1.62 (0.44)	1.58 (0.43)	1.73 (0.48)	1.53 (0.41)	*F*(3, 294) = 2.52, *p* = 0.058

^1^The German school system has three type of school degrees; lowest = ‘Hauptschulabschluss’, which can be acquired after the 9th, middle = ‘Realschulabschluss’, which can be acquired after the 10th, and high = ‘Abitur’, which can be acquired after the 12th or 13th school year.

^2^Scores can range from 1 to 4, which indicate the average lifetime frequency of psychotic (positive or negative symptoms) symptoms (1 = never, 2 = sometimes, 3 = often, 4 = nearly always). For reference: Mossaheb et al.^19^ report means (SD) for frequency on the positive symptom dimension individuals with ultra-high-risk for psychosis (n = 84) vs. without risk (i.e., healthy controls; n = 81): 1.9 (0.5), [CI 1.71–2.02] vs. 1.6 (0.4), [CI 1.47–1.70].

### Sample

Initially, *N* = 401 individuals started the survey. Of those, *n* = 76 quit during the sociodemographic assessment, *n* = 24 lacked pre-test data, and *n* = 6 lacked post-test data. These *n* = 106 cases were excluded from the analyses, resulting in a final sample of* N* = 295. Of these, some participants had incomplete post-test data (*n* = 10 missing digit span, 5 missing n-back, and *n* = 8 missing the qualitative assessments [sound rating]).

For detailed information and inferential statistics comparing the groups at baseline see Table 2. The participants were in their middle to late twenties on average and there were in tendency more males than females. Net income was mostly reported to be in the lowest category (i.e., < 1.250€, 40–50% of participants of all groups), but also between 5 and 20% of participants did not wish to reveal their monthly net income. Positive symptom frequency levels did not differ significantly between the groups, albeit there were relatively marked descriptive differences (*p* = 0.058). The values were mostly similar and within a confidence interval range that has previously been reported for healthy individuals^19^. Due to the trend-level nature of the differences in positive symptom frequency, we decided to repeat the main analyses, controlling for this variable as covariate in the repeated measures ANOVAs.

### Measures

For all mood and the paranoia scales, item scores were computed (i.e., summing up responses on all items and dividing this by the number of items). This way, the interpretation of scores is facilitated, as it corresponds to the Likert-scale of the respective measure.

#### Psychosis liability

Psychosis-liability or sub-clinical psychosis levels was assessed using the Community Assessment of Psychic Experiences (CAPE)^19^, in its German version, to assesses lifetime positive, negative and depressive symptoms (http://www.cape42.homestead.com/index.html). The CAPE, including the German version, has been validated extensively^17^. Items refer to the lifetime prevalence of specific symptoms, rated on an ordinal response scale for frequency (categories: 1 = ‘never’, 2 = ‘sometimes’, 3 = ‘often’, 4 = ‘nearly always’). The total scale consists of 42 items, whereby the positive symptom scale includes 20 (e.g., ‘Do you ever feel as if things in magazines or on TV were written especially for you?’), the negative symptom scale 14 (e.g., ‘Do you ever feel that your mind is empty?’), and the depressive symptom scale 8 items (e.g., ‘Do you ever feel like a failure?’). To test for comparative baseline levels across all groups in psychosis liability, mean frequency scores for the positive symptom subscale was used, for which Mossaheb and colleagues have provided descriptive data for individuals with ultra-high risk for psychosis (n = 84) vs without risk (i.e., healthy controls; n = 81)^19^. The positive dimension (frequency) of the CAPE had excellent internal consistency in the present sample, with Cronbach’s α = 90.

#### Mood and paranoid symptoms

Mood was assessed with the State Trait Anxiety Depression Inventory (STADI)^27^. The scale contains 40 items, whereby the same 20 items are once presented in trait and once in state format. Only the latter was used in the present study. The scale differentiates between depression (low euthymia [inverted items], dysthymia) and anxiety (hyperarousal and worry), whereby each of the subscales is assessed by 5 items. The response format is a 4-point Likert (1 = ‘not at all’, 4 = ‘strongly applies’). Internal consistency (Cronbach’s *α*) at pre-test was good both for the state anxiety (0.85) and depression (0.86) scales.

Paranoia was assessed with a brief, change sensitive state version of the paranoia checklist, which has been validated and comparable to the long, state adapted 18-item version^17^. The scale comprises 3 statements (e.g., ‘I need to be on my guard against others’, ‘Strangers and friends look at me critically’, ‘People try to upset me’), rated on an 11-point Likert-scale (each from 1 to 11) for the degree of agreement to the statement, associated distress and conviction, at present. The latter two categories were only presented if the rating of agreement to the statement was > 1 (which accordingly often results in a large amount of missing data). In the present study, only agreement was evaluated. Internal consistency at pre-test was acceptable with Cronbach’s α = 0.78.

#### Cognition

To assess digit span cognitive performance, both the forward and backward version were used, as available in Inquisit 5^26^ [retrieved from https://www.millisecond.com] which is based on the original task reported by Woods et al.^28^. Two parameters are recommended for evaluation: the two-error maximum length (TE_ML) and the maximum length recalled (ML). The two-error maximum length is defined as the last digit span a participant gets correct before making two consecutive errors while the maximum length is the digit span that a participant recalled correctly during all trials irrespective of the number of errors in-between. Starting with a successive visual presentation of 3 digits, the participants need to correctly recall a by 1 digit increasing sequence of digits and reproduce it by clicking on the correct digits in correct order. After two wrongly recalled sequences of the same length, the digit span is decreased by 1 digit until the digit span length again reaches the starting point of 3. The total amount of trials is 14 making the shortest span possible 3 digits long and the longest span 16 digits long. The participants were explicitly reminded not to use any memory assisting methods such as paper and pencil. The dual n-back task, also available in the Inquisit 5^26^ (retrieved from https://www.millisecond.com) was assessed. The task is based on the original work by Jaeggi et al.^29^. It consists of 4 experimental blocks demanding 2-back and 3-back level performance. While performing the task, subjects pay attention to their computer screen while also listening to a computer audio. On each trial a blue square appears in one out of eight grid-like locations around a central fixation cross, while at the same time a (German) letter is presented via the headphones. In the 2-back block condition, the subjects are instructed to press the “A” button on their keyboard when the current square position matches the square position from two trials before. Subjects are also instructed to press the “L” button on their keyboard if the spoken letter matched the letter two trials before. The same instruction, but having to match stimuli 3 trials back, is provided for the 3-back condition. In the present study, participants trained each condition once, and then went on with the experimental blocks. The performance parameter was the so-called *d* prime value calculated as the proportion of ((visual_TotalHits − visual_TotalFA) + (auditory_TotalHits − auditory_TotalFA)/2)/number of total experimental blocks. The highest possible *d* prime (greatest sensitivity) was 6.93 and the lowest was 0. Visual hits are defined as correct responses with respect to the location of the square and auditory hits are defined as the correct responses with respect to the spoken letter. Visual false alarms (FA) are defined as responses in the absence of a target in the visual domain, thus with respect to the location of the square and auditory false alarms are responses in the absence of a target in the auditory domain, thus with respect to the spoken letter.

#### Soundscape perception

The participant’s perception of the soundscapes was assessed using a one item questionnaire per dimension (diversity/monotony, pleasantness, and beauty). Participants were asked to report on a 0 to 100 visual scale how diverse/monotone, beautiful, and pleasant they had perceived the soundscape they had listened to during the experiment. The items have been formulated by the authors themselves while the use of an aesthetic rating of the soundscapes per se was a replication from the van Hedger et al.^16^ study where we exchanged the “like–dislike” affective response with a more detailed aesthetic rating splitting the response up into a pleasantness and a beauty dimension. The dimension of diversity/monotony has been to perform a manipulation check on diversity for the soundscapes used in the present study.

### Stimuli

The soundscapes for all four categories have been generated in the same way. Single sound snippets were gathered and then adapted and merged within the audio software *Steinberg Cubase10*. An exemplary visualization of the resulting soundscape can be seen in the Supplementary Material (see Supplementary Fig. 1). For the nature category a database of birdsong recordings (https://www.xeno-canto.org/explore/region) from a central European origin was used. For the low diversity birdsong condition, eight recordings from the same two species were used (common chiffchaff & wood warbler). For the high diversity birdsong condition, the same approach was chosen, but recordings from eight different bird species were used to create the soundscape (garden warbler, honey buzzard, woodlark, Eurasian sparrow hawk, coal tit, greenshank, common crane, and black woodpecker). In both birdsong conditions, additionally subtle water and wind sounds were played in the background, to create a constant auditory experience. For the traffic noise conditions, sound snippets from eight car recoding’s (https://freesound.org/search/?q=city) were used for the low diversity traffic noise condition while audio-snippets from eight diverse sources of noise pollution associated with the city were used for the high diversity traffic noise condition (ambulance siren, construction, trucks, train, motorcycle, airplane, bus and fire-fighter siren). In both traffic noise soundscapes, a constant subtle traffic flow was audible in the background.

To ensure that all soundscapes were perceived with a similar loudness level, all soundscapes were engineered to have a similar loudness value. The loudness values from all four conditions range between 19.4 and 27.8 loudness units relative to full scale (LUFS). All soundscapes had a duration of 6 min. Prior to the experiment the soundscapes have been presented to a small set of pilot participants rating the similarity of the audio level ensuring a comfortable audio level across all conditions. As a result, at the beginning of the experiment, participants were instructed to set their headphone loudness level to 80%. Soundscapes can be accessed openly via this link https://osf.io/4y3vh/.

### Statistical analyses

Analyses were run in SPSS 27 (IBM Corp., 2020). To test the differences of all measures at baseline, several univariate analyses of variance (ANOVA) were run. In order to test the effects of high vs. low diverse traffic noise vs. birdsong soundscapes on mood, paranoia, and cognition, repeated measures analyses of variance (ANOVA) were run testing for a 2 (*timepoint*: pre vs. post) × 2 (*soundscape type*: birdsong vs. traffic noise) × 2 (*diversity*: low vs. high) interaction effect. The analyses were once run with all participants, and then only with those who entered at least one of the digits (control of compliance of listening to audio, see “Study procedure” section) correctly, to check for the robustness of findings. In order to further check for the robustness of effects on mood and cognition repeated measures ANOVAs were run controlling for baseline sample differences on sample characteristics or outcomes (i.e., state paranoia, age and positive symptoms) as covariates. To check the robustness of effects on paranoia a univariate analysis of covariance (ANCOVA) with paranoia at baseline as covariate and post-test paranoia as outcome, predicted by *type* and *diversity* as factors, was computed. Significant interactions (i.e., of interest were the *type* × *time* and *type* × *diversity* × *time*) interactions identified for any of the outcomes were followed up by subsequent detailed post-hoc-tests. To explore mean differences between the qualitative ratings of soundscapes (i.e., beauty, pleasantness, and monotony vs. diversity), a one-way multivariate analysis of variance (MANOVA) was conducted. In case of significant omnibus tests indicating global differences across the qualitative sound rating dimensions, follow-up between group t-tests were conducted. Due to the exploratory nature of the study, no *p*-level correction was applied.

The partial eta squared effect size was used to interpret the ANOVA based analyses, with the corresponding rule of thumb defining *η*^2^ = 0.01 as a small effect size, *η*^2^ = 0.06 as a medium effect size and *η*^2^ = 0.14 as a large effect size^30^. Cohen’s d effect size was used to interpret post-hoc test effect sizes, with the corresponding rule of thumb defining a value of ≥ 0.2 as a small effect size, a value of ≥ 0.5 as a medium effect size and a value of ≥ 0.8 as a large effect size. The criteria for interpreting the effect size for Hedge’s g stem from the corresponding rule of thumb with the same definition^30^.

### Ethics statement

All procedures performed in studies involving human participants were in accordance with the ethical standards of the institutional and/or national research committee and with the 1964 Helsinki Declaration and its later amendments or comparable ethical standards. Informed consent was obtained from all participating subjects. The experimental protocol was approved by the ethical committee from the University Clinic Hamburg Eppendorf.

## Supplementary Information

Supplementary Information.

## Data Availability

The datasets generated during and/or analyzed during the current study are available from the corresponding author on reasonable request.
