# Supplementary material for: Birdsongs alleviate anxiety and paranoia in healthy participants
Source: Sci Rep. 2022 Oct 13;12:16414. doi: 10.1038/s41598-022-20841-0 (PMC9561536; doi:10.1038/s41598-022-20841-0)
Supplement: Supplementary file 1 — Supplementary Information. [file 41598_2022_20841_MOESM1_ESM.docx]

**Supplementary Material**

**Supplementary Table 1.** Descriptive data for the qualitative sound ratings

|  |  | **Monotony/**  **Diversity** | **Beauty** | **Pleasantness** |
| --- | --- | --- | --- | --- |
| **Groups** | **n** | **Mean (SD)** | | |
| Diversity: low | 144 | 49.40 (26.03) | 50.27 (31.37) | 52.86 (27.67) |
| Diversity: high | 138 | 57.35 (25.57) | 51.06 (30.61) | 54.04 (29.99) |
| Type: city noise | 149 | 52.56 (27.40) | 32.84 (27.08) | 36.50 (23.93) |
| Type: birdsongs | 133 | 54.11 (24.55) | 70.62 (21.38) | 72.41 (20.86) |
| City noise low diversity | 83 | 47.95 (28.76) | 36.22 (29.58) | 40.16 (24.62) |
| City noise high diversity | 66 | 58.36 (24.59) | 28.59 (23.08) | 31.91 (22.37) |
| Birdsongs low diversity | 61 | 51.38 (21.84) | 69.39 (22.47) | 70.15 (21.65) |
| Birdsongs high diversity | 72 | 56.42 (26.56) | 71.65 (20.52) | 74.32 (20.12) |

**Note.** Inferential statistics concerning significant differences between these groups/ factors are provided in the main paper (Table 1, p. 7)

**Supplementary Table 2.** Descriptive pre-post data of main outcomes for all conditions

**Supplementary Table 3.** Paired t-test statistics for changes within groups in mood and paranoia

# Supplementary Figure 1.


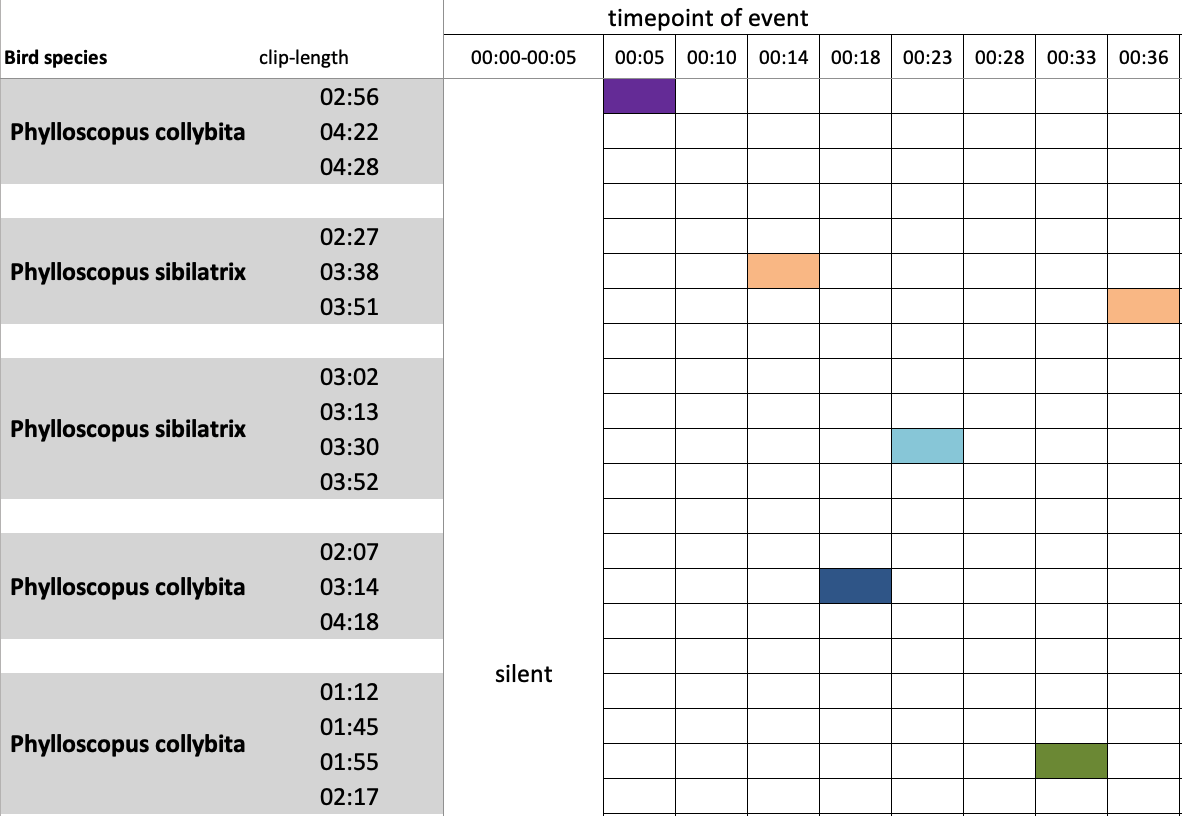


Supplementary Figure 1. Depicted here is an example on how the low diversity birdsong condition was generated. On the left the participating bird species are listed and on the right a flow of events is shown
